# Predicting Binding Free Energy Change Caused by Point Mutations with Knowledge-Modified MM/PBSA Method

**DOI:** 10.1371/journal.pcbi.1004276

**Published:** 2015-07-06

**Authors:** Marharyta Petukh, Minghui Li, Emil Alexov

**Affiliations:** 1 Computational Biophysics and Bioinformatics, Department of Physics, Clemson University, Clemson, South Carolina, United States of America; 2 National Center for Biotechnology Information, National Library of Medicine, National Institutes of Health, Bethesda, Maryland, United States of America; University of Maryland, UNITED STATES

## Abstract

A new methodology termed Single Amino Acid Mutation based change in Binding free Energy (SAAMBE) was developed to predict the changes of the binding free energy caused by mutations. The method utilizes 3D structures of the corresponding protein-protein complexes and takes advantage of both approaches: sequence- and structure-based methods. The method has two components: a MM/PBSA-based component, and an additional set of statistical terms delivered from statistical investigation of physico-chemical properties of protein complexes. While the approach is rigid body approach and does not explicitly consider plausible conformational changes caused by the binding, the effect of conformational changes, including changes away from binding interface, on electrostatics are mimicked with amino acid specific dielectric constants. This provides significant improvement of SAAMBE predictions as indicated by better match against experimentally determined binding free energy changes over 1300 mutations in 43 proteins. The final benchmarking resulted in a very good agreement with experimental data (correlation coefficient 0.624) while the algorithm being fast enough to allow for large-scale calculations (the average time is less than a minute per mutation).

## Author Summary

Developing methods for accurate prediction of effects of amino acid substitutions on protein-protein affinity is important for both understanding disease-causing mechanism of missense mutations and guiding protein engineering. For both purposes, there is a need for accurate methods primarily based on first principle calculations, while being fast enough to handle large number of cases. Here we report a new method, the Single Amino Acid Mutation based change in Binding free Energy (SAAMBE) method. The core of the SAAMBE method is a modified molecular mechanics Poisson-Boltzmann Surface Area (MM/PBSA) method with residue specific dielectric constant. Adopting residue specific dielectric constant allows for mimicking the effects of plausible conformational changes induced by the binding on the solvation energy without performing computationally expensive explicit modeling. This makes the SAAMBE algorithm fast, while still capable of capturing many of the explicit effects associated with the binding. The performance of the SAAMBE protocol was tested against experimentally determined binding free energy changes over 1300 mutations in 43 proteins and very good correlation coefficient was obtained. Due to its computational efficiency, the SAAMBE method will be soon implemented into webserver and made available to the computational community.

This is a *PLOS Computational Biology* Methods paper.

## Introduction

One of the most essential properties of all living organisms is the ability to conduct comprehensive “communication” between its individual components. This includes signal transduction, immune system operation, inhibition or activation of particular functions, assembly of macromolecular structures into molecular machines (such as ATPase), and much more. At the molecular level such communications are carried out via macromolecular binding [1,2]. The molecular recognition is affected by multiple factors such as concentration and compartmentalization of the macromolecules, their shapes, charge distribution, conformational flexibility, physico-chemical properties of the interfaces and many others [3–11]. Any change of these characteristics could alter the wild type protein binding and therefore might affect the function of macromolecules. While some of abovementioned factors (macromolecular and salt concentrations, pH and temperature of the media, etc.) are results of the cellular function, other characteristics (physico-chemical properties of interfaces, protein charge distribution, etc) are largely determined by protein amino acid sequence and structure. Because of that, any alteration of the protein primary structure (insertion, deletion or amino acid substitution) may have an effect on macromolecular recognition. Having in mind that *in vivo* interactions occur in the crowded cellular environment, mutations may not only impact binding affinity but also could perturb protein interaction networks resulting in a loss or gain of interactions. Such changes in binding and interactions are frequently implicated in diseases and understanding of their molecular mechanisms is crucial for deciphering the origin of diseases. In particular, the effect of mutations on binding free energy (binding affinity) is considered to be an important component of the overall disease effect [12].

The effect of missense mutations on protein-protein complex formation can be experimentally assessed by various techniques such as isothermal titration calorimetry [13], FRET [14], surface plasmon resonance [15], and many others (see review [16]). However they are time-consuming, expensive to carry out and cannot be applied on a large scale. Despite such limitations, investigators have performed many mutagenesis experiments in the past to determine the effects of point mutations on binding free energy. The results reported in the literature were compiled into useful databases, the most prominent one being Skempi database [17]. Although most of these experiments were carried out on protein complexes that were either easy to manipulate biochemically or were of particular interest for the molecular biology community at that time, still such databases can be considered representative for any other interactions since the biophysical principles governing the binding should be universal. Therefore, these experimentally determined binding free energy changes caused by point mutations can serve as an ultimate benchmark for computational methods aiming at *in silico* predictions.

Obviously, large-scale studies of the effects of mutations on protein-protein binding require computational approaches. Roughly speaking, the existing computational methods can be divided into two main categories: sequence-based and structure-based approaches. The main advantage of sequence-based approaches is that they are fast, but the techniques used for the predictions strongly depend on the training set of data [18] and may be over-fitted [19]. On the other part of the spectrum are structure-based approaches, many of them providing a qualitative estimate (beneficial/neutral/deleterious) of the changes in binding affinity upon mutations [20]. Multiple approaches in this category utilize different scoring schemes, solvent models (implicit/explicit models), number of representative structures used in the analysis, Monte Carlo and molecular dynamics sampling methodologies, etc. (for some examples see [21–29]). Among the structure-based approaches the most rigorous (theoretically exact) methods are the free energy perturbation (FEP) and thermodynamic integration (IT) methods [30]. However, they require intensive calculations and cannot be applied for large-scale modeling (see review [31]).

Among the structure-based methods, the Molecular Mechanical Poisson-Boltzmann (Generalized Born) / Surface Accessible (MM/PB(GB)SA) approach [32–34] represents a reasonable balance between computational time and details of the modeling. In this approach the binding free energy is calculated as a linear combination of potential energies such as molecular mechanics, polar and non-polar solvation energies. Similarly one can construct a function made of linear combination of weighted terms, either statistically or empirically delivered, to predict binding free energy and the change of it due to mutations [21,35]. Hybrid approaches do exist as well [24,25]. Some of these approaches emphasize on the importance of taking into account structural ensembles in the modeling [25], others on the role of water phase and solvation energy [24].

In this paper we introduce a new methodology termed Single Amino Acid Mutation based change in Binding free Energy (SAAMBE), which takes advantage of both approaches: sequence- and structure-based methods. It utilizes MM/PBSA approach along with an additional set of statistical terms delivered from statistical investigation of the physico-chemical properties of protein complexes. The new method was tested against more than 1300 mutations in 43 proteins and resulted in a very good agreement with experimental data (correlation coefficient 0.624) while being fast enough to allow for large-scale calculations (the average time is less than a minute per mutation).

## Results and Discussion

Our goal is to create a fast and accurate method to predict the changes of binding free energy of the protein-protein complex caused by single point mutations. The approach combines MM/PBSA method with knowledge-based terms. The optimal parameters of the weights in linear formula were obtained via multiple linear regression analysis against experimental values of ΔΔ*G* in tDB. Below we describe the investigations done to test the sensitivity of the protocol against various parameters, to obtain the optimized weight coefficients for the SAAMBE formula and to benchmark the protocol against experimental data.

### Optimizing the parameters of MM/PBSA-based component of SAAMBE method

#### Optimization of NAMD protocol

We tested different parameters for the NAMD-based simulation protocol in order to select the optimal values and modeling strategies.

- Structure relaxation. It is anticipated that the binding is associated with small or large conformational changes and these conformational changes may not be the same for the WT and MT proteins. Typically these conformational changes are modeled by carrying out molecular dynamics (MD) simulations with various lengths of simulation time and collecting representative snapshots for further analysis. Following this approach we tested MM/PBSA performance by subjecting the WT and MT complexes and separated monomers to energy minimization (200, 500, 1000, 2000, 5000, 10000, 15000 and 40000 steps) followed by MD simulations (10000, 15000 and 40000 steps, 2 ps per step) at room temperature. However, the benchmarking against experimental data indicated that the MD simulations protocol results in worse (compared with simulations without MD) correlation of SAAMBE predicted change of the binding free energy with the experimental data. Because of that, MD simulations are not included in the SAAMBE protocol.
- Degree of structural refinement. While structural relaxation via MD simulations was shown not to improve the correlation between SAAMBE calculated ΔΔ*G* values and experimental data, still structures must be energy minimized to obtain the MM/PBSA energy components. The energy minimization (structural refinement) was done with the minimization module of NAMD. We tested a broad spectrum of the number of equilibration steps to minimize the WT and MT complexes with implicit solvent model for all entries in the tDB. The structures obtained with 5000-steps minimization resulted in the best correlation between SAAMBE predicted and experimental values of the changes of the binding free energy. Minimizations with smaller number of steps (we tried 200, 500, 1000 and 2000) were shown to be insufficient for the structural refinement, probably because of the large size of the most of the complexes in the tDB. On the other hand, using a larger number of steps (we tried 10000, 15000, 40000) reduced the agreement of the calculated results with experimental data as well.
- Dielectric constant (ε) of the protein for the GB model in NAMD. The energy minimization was done by modeling the water phase with GB model implemented in NAMD. It allows protein dielectric constant to be selected. Among different dielectric constants (we tried 1, 2, 4, 8, 12) we selected ε = 1 since it was shown to result in best correlation between SAAMBE predicted and experimental values of the changes of the binding free energy.

Thus, the SAAMBE protocol subjects the structures of WT and MT complexes to 5,000-step energy minimization with GB implicit solvent. Dielectric constant is 1. The *IE* and *VE* energies are delivered with these parameters from standard NAMD output. It should be mentioned that we also tried structural relaxation and refinement on separated monomers, but the results were worse. Because of that, the SAAMBE protocol keeps the structures of the monomers as they are in their bound form.

#### Choosing dielectric constants for electrostatic energies (DelPhi)

**Fig 1:**
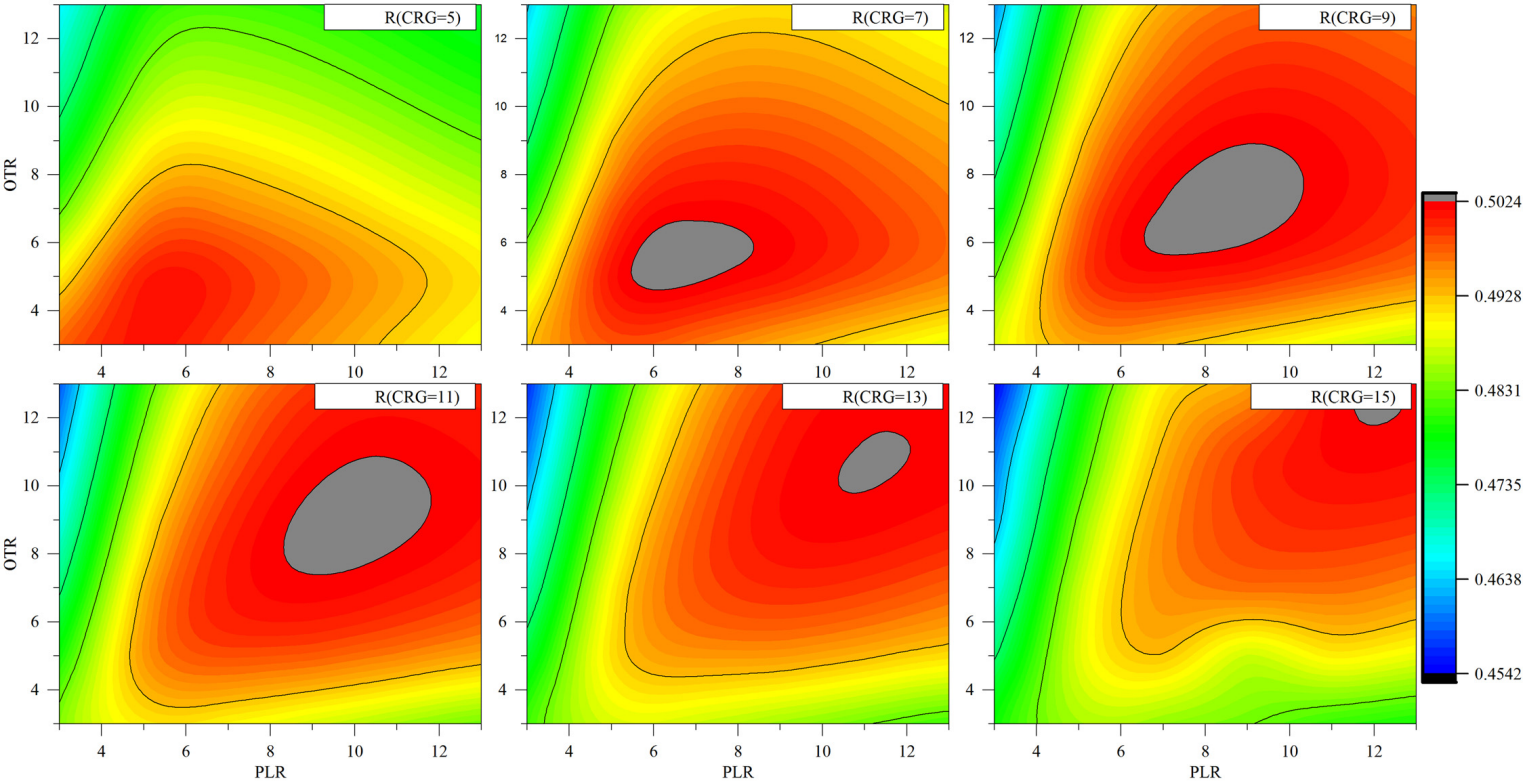
The effect of dielectric constant variation for charged, polar and other residues in calculations of EE and SP on the correlation coefficient between experimental and calculated values of the change in binding free energy for the tDB. Only EE, VE and SP components were taken into account for the multiple linear regression analysis.

Since structural refinement with NAMD was done in implicit solvent model with dielectric constant 1, it is expected that the same value should be used to calculate the electrostatic components of the energy. However, initial testing showed that the obtained correlation of SAAMBE predicted energy changes and experiments is not impressive. This combined with our previous work on predicting folding free energy changes [36], we decided to test the possibility that better correlation can be obtained if amino acids with different physico-chemical properties are modeled with different dielectric constants. Previous investigations indicated that charged and polar amino acid should be assigned relatively large dielectric constant as compared with hydrophobic groups [36]. However, the work was done for predicting folding free energy changes and the results may not be directly transferrable to model the changes of the binding free energy. In SAAMBE protocol, we assume that there are three groups of residues with specific dielectric constants ε_1_, ε_2_ and ε_3_ for charged, polar and other groups, respectively (see Method section). We varied systematically the dielectric constant for charged groups from 5 to 15, for polar from 3 to 13 and other residues from 3 to 13 with a step of 2. Then multiple linear regression analysis was performed for SAAMBE formula containing *EE*, *VE* and *SP* components only. This was done for computational efficiency only. Fig 1 shows contour maps of the correlation coefficients for fixed ε_1_ of charged residues and varied ε_2_ of polar residues (on the *x-axis*) and ε_3_ for other types of residues (on the *y-axis*). The grey color represents the area with the maximum correlation coefficient, while the black one—its minimum for given combination of dielectric constants. From Fig 1 one can see that the area with maximum correlation coefficient increases with the increase of dielectric constant of the charged residues, reach its maximum at ε_1_ = 9 and then decreases. The correlation coefficient has the highest value when the ε_2_ for the polar residues is 8 and ε_3_ for other types of residues is 7. Thus SAAMBE protocol uses dielectric constants of 9, 8, and 7 for charged, polar and other amino acids, respectively, to calculate the *SP* energy component. The *EE* component is calculated with the lowest dielectric constant, ε = 7, for the entire protein and protein complex.

### Optimizing the parameters of knowledge-based component of SAAMBE protocol

As described in the method section, several knowledge-based terms were tested to improve the correlation between predicted and experimental ΔΔ*G*. One of these terms was added in the SAAMBE formula to mimic the effect of the change of conformational entropy caused by mutations (*ΔΔS* term). Others—because of our previous work as *Interface* ^*MT*^ term in Eqs (4 and 8) [24]. The third set of terms was introduced in SAMMBE formula due to extensive testing of various physico-chemical characteristics as hydrophobicity *(ΔΔHYDR*), hydrogen bonds *(ΔHB)* and normalized change of the interface area caused by mutations ($\frac{\Delta \Delta SASA}{Interface^{MT}}$). It is understood that there is an overlap between some of these terms and the terms within MM/PBSA-based method, and between themselves alone as well. Hydrogen bond change is partially accounted for in MM/PBSA algorithm via the electrostatic energy term. The *Interface* ^*MT*^ and $\frac{\Delta \Delta SASA}{Interface^{MT}}$ are also related. However, the overlap is not complete as shown by the provided p-values (Table 1). The functional form of knowledge-based terms was optimized by trying various forms as explained in the method section. Their optimized forms are the one shown in Eqs (8)–(12).

**Table 1:** The weights of energy terms in calculating binding free energy and parameters of linear function between experimental and predicted ΔΔ*G*.

	tDB_small		tDB_large		tDB	
	weight	p-value	weight	p-value	weight	p-value
Free	0.74345	2.61E-06	2.68491	0	1.81729	0
ΔΔ*EE*	0.24695	1.83E-07	0.38921	0	0.39117	0
ΔΔ*VE*	0.1405	8.99E-06	0.18347	6.66E-16	0.18732	0
ΔΔ*SP*	0.26	2.77E-06	0.44347	2.22E-16	0.43118	0
ΔΔ*SN*	0.00354	2.90E-02				
ΔΔ*S*	0.17197	9.80E-02	0.1848	9.72E-03	0.20841	2.00E-04
ΔΔ*HYDR*			0.55761	2.10E-05	-0.6731	1.48E-10
*Interface*	1.67E-04	1.22E-02	6.37356E-04	3.04E-06	4.64209E-04	3.64E-10
ΔΔ*ME*	0.03538	1.57E-06	0.053	1.63E-05	0.06648	0
Δ*HB*			0.03585	9.24E-02		
**$\frac{\Delta \Delta ASA}{Interface}$**	7.75803	2.79E-03			9.82407	1.18E-05
**Ncases**	612		714		1326	
Slope	-2.3058E-5				-7.0929E-07	
Y-int	1				1	
**Correlation**	**0.624 (0.716^±2SD^, 0.603^CV^)**				**0.575**	

For all weights p<0.1. Data in brackets is for tDB within 2SD, and the one based on 5-fold cross validation.

### Statistical analysis of experimental data

**Fig 2:**
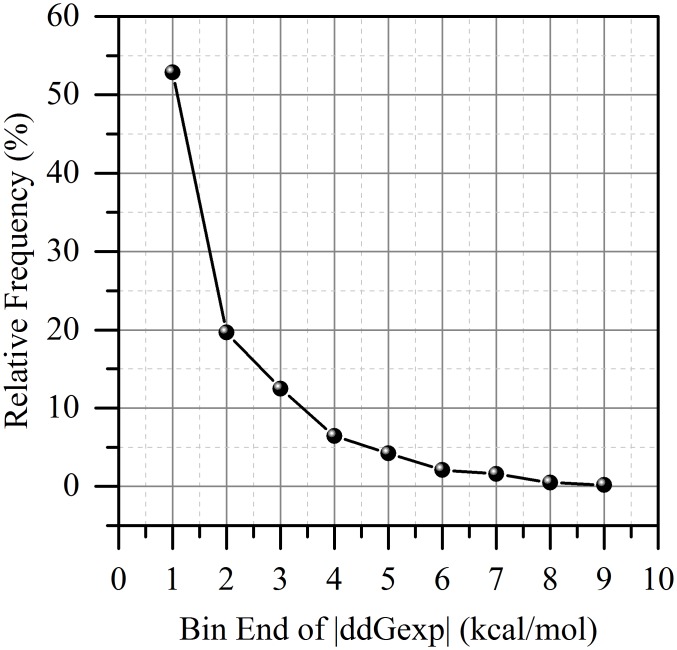
The distribution of the absolute values of the experimental ΔΔ*G* in sDB.

The experimentally measured changes of the binding free energy caused by mutations vary from zero to very large positive values (+8.803) and very small negative values (-3.786). It can be anticipated that there may be some structural or sequence characteristics associated with the magnitude of the binding free energy change. To test such a possibility, we first provide the distribution of the absolute changes of experimental binding free energy in sDB dataset (Fig 2). It can be seen that the cases with absolute binding free energy change of less than 1kcal/mol account for about 50% of the cases. Therefore we chose to split the whole database into two sets with similar number of entries: one set with small effect (|ΔΔ*G*|<1kcal/mol); and another with large effect (|ΔΔ*G*|≥1kcal/mol).

The next step was to determine the probability of mutations to cause “small effect” or “large effect” depending on two characteristics: amino acid type and location of the mutation site at the interfacial regions. With regard to amino acid types, we will consider WT and MT separately as explained below. With regard to interfacial location, we use the definitions provided in the Method section (COR, SUP, RIM, INT and SUR).

**Fig 3:**
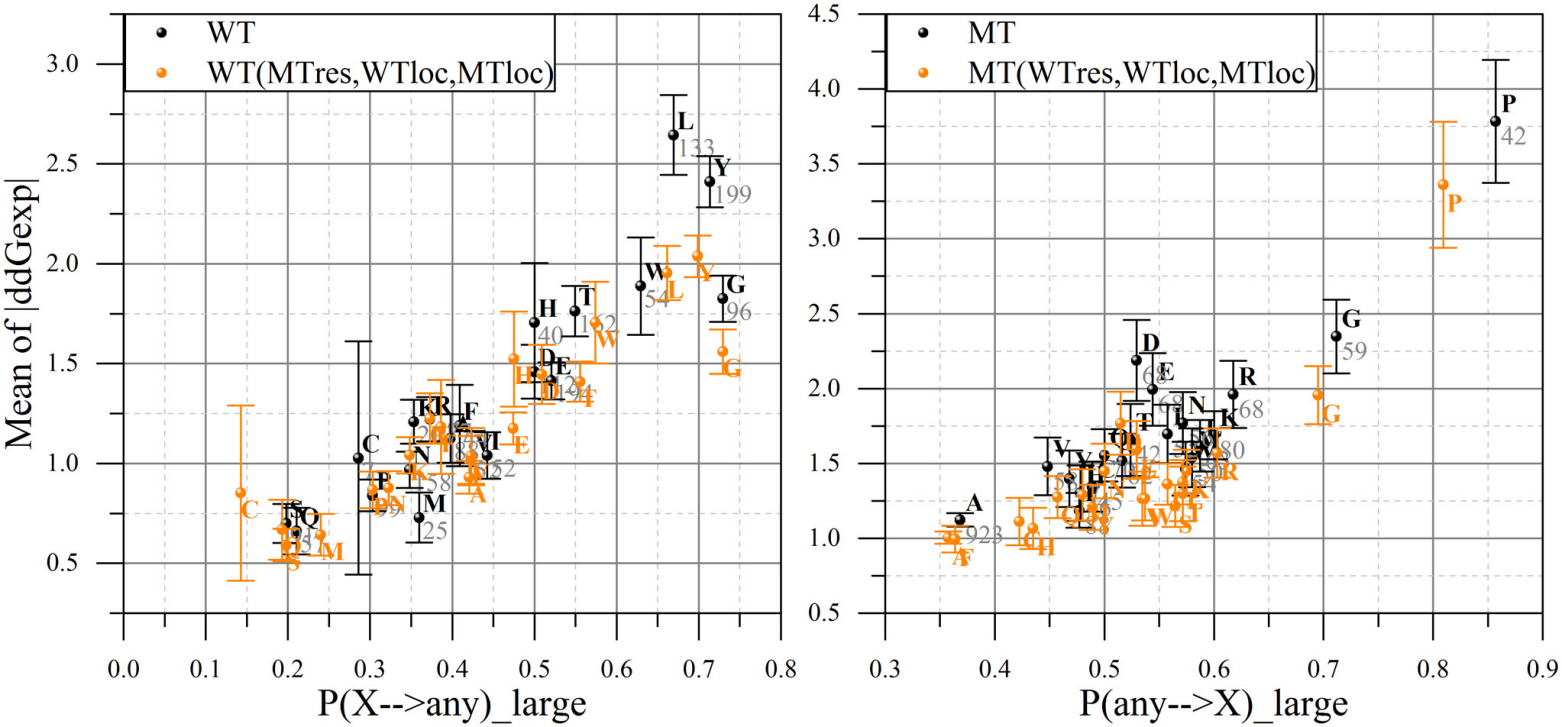
Distribution of residue types (being as WT, left panel; being as MT, right panel) by "small/large effect" regions of experimentally obtained change in binding free energy in sDB. On the *x-axis*: the probability of the particular type of residue substitution (WT on left panel, MT—on the right one) to result in a large change in binding free energy. On the *y-axis*: the averaged absolute value of experimental ΔΔ*G* provided with standard error of mean at an error bar and the total number of cases across whole sDB. The actual data is presented in black color, while the orange one is based on the weighted distribution of |ΔΔ*G*| (see text for details).

Furthermore we collect all available substitutions M of a given type *X* → *any* residue, where X is a particular amino acid (for example, Ala, Arg, etc). Then we calculate the mean and variance of experimental change of the binding free energy for these M cases. In addition, we introduce an estimation of the probability (P) of mutation type *X* → *any* to cause large effect by:

$$\text{P}(\text{X}\to \text{any})=\frac{{\text{M}}_{\text{large}}}{\text{M}}$$ (1)

where *M*_*large*_ is the number of cases within *M* subset for which the absolute change of the binding free energy is larger than 1kcal/mol (large effect) (Fig 3, left panel). Similarly we perform the same analysis for substitutions of (*any* → *X*) and define the corresponding probabilities *P*(*any* → *X*) (Fig 3, right panel).

**Fig 4:**
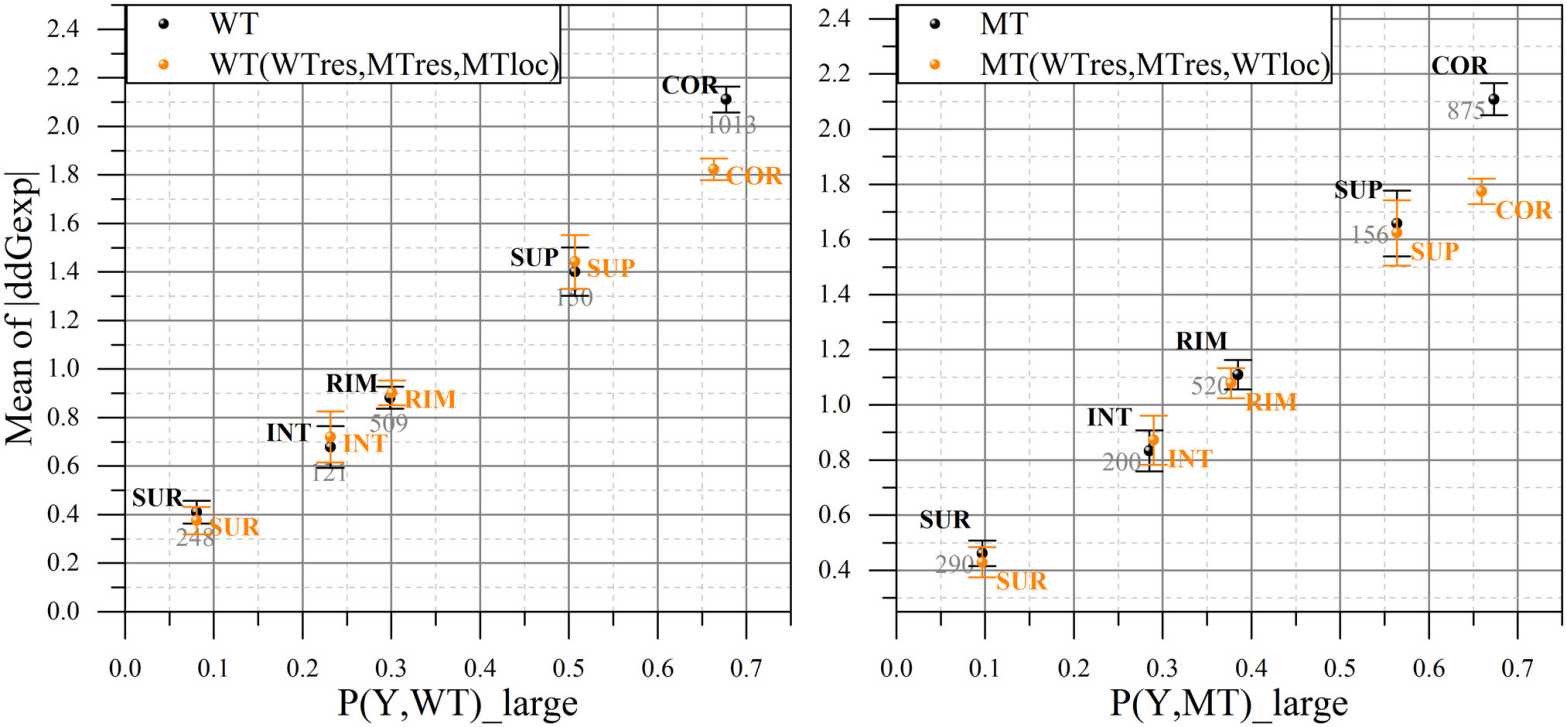
Distribution of mutated residue location (WT, left panel; MT, right panel) by "small/large effect" regions of experimentally obtained change in binding free energy in sDB. On the x-axis: the probability of the WT (left panel) and MT (right panel) residues being in the given location cause large change in binding free energy. On the y-axis: the averaged absolute value of experimental ΔΔ*G* provided with standard error of mean at an error bar and the total number of cases across whole sDB. The actual data is presented in black color, while the orange one is based on the weighted distribution of |ΔΔ*G*|.

With respect to mutation site location, we select all available cases K for which the mutation site in the WT is located at Y, where Y is either COR, SUP, RIM, INT or SUR. Then we define a probability of mutations within K to cause large effect as:

$$\text{P}(\text{Y},\text{WT})=\frac{{\text{K}}_{\text{large}}}{\text{K}}$$ (2)

where *K*_*large*_ are the cases experimentally found to result in absolute binding free energy change larger than 1kcal/mol (Fig 4, left panel). Since mutations involve amino acids with different side chain length and MT and MT structures are subjected to energy minimization, it is quite likely that mutation site location is different in MT compared with WT. For this reason, the same analysis is done for the MT and the corresponding probabilities are defined as *P*(*Y*, *MT*) (Fig 4, right panel).

Fig 3 indicates that there is a tendency for some types of substitutions to cause small, while other to cause large effects on the binding free energy. It can be seen that most of substitutions for Tyr and Gly in WT (P > 0.7) cause a big change of binding free energy. Consistent with our previous work [24], mutations to Pro and Gly also often (P > 0.7) cause large changes in binding free energy. These results are not surprising since Tyr is a bulky aromatic polar residue. Two effects may be involved in stabilization of the WT structure by this amino acid: formation of hydrogen bond with other charged/polar residues and noncovalent interactions with aromatic rings of other residues such as Trp and Phe, known as “stacking effect”. Two other residues, Pro and Gly, are considered to be special in terms of their physico-chemical characteristics. Although both of them are most often found in a coil rather than in a sheet or strand, they perform different structural roles. Namely, Gly makes the secondary structure more flexible, while Pro tends to rigidify it. Pro is also a well-known secondary structure element breaker—it forms a turn when being introduced in a helix or a strand.

The mutation site location also shows distinctive trend (Fig 4). There is almost linear correlation between the mean of the absolute binding energy change and the probability (Eq (2)). Thus, the probability of a mutation located at mutations site, both in WT and MT, to cause large change of the binding free energy gradually increases: SUR → INT → RIM → SUP → COR.

These observation and the corresponding probabilities can be used to guide SAAMBE predictions. However, before proceeding further with these possibilities, we should analyze the results presented in Figs 3 and 4. Essentially, four “flags” were identified with four associated probabilities: residue type in WT and *P*(*X* → *any*), residue type in the MT and *P*(*any* → *X*), mutation site in WT and *P*(*Y*, *WT*) and in MT and *P*(*Y*, *MT*). Therefore, a consensus scheme must be developed in order to incorporate these quantities into the SAAMBE algorithm. Further refinement of the classification scheme was done by altering the associated *ΔΔG*_*i*_ for cases for which there is no agreement between the four “flags”. For example, if a given mutation Q → P in “k” case in sDB with experimentally determined |ΔΔ*G*_*k*_| = 10kcal/mol and the mutation sites are in COR in WT and in SUP in MT. From Figs 3 and 4 the corresponding probabilities of causing strong effect are: *P*(*Q* → *any*) = 0.2, *P*(*any* → *P*) = 0.86, *P*(*COR*, *WT*) = 0.68 and *P*(*SUP*, *MT*) = 0.56. Based on these probabilities, one expects that any mutation from Q will have little chance to cause strong effect (*P(Q* → *any)* = 0.2), but the specific case of Q → P was experimentally found to result in a large change (|ΔΔ*G*_*k*_| = 10kcal/mol). It can be speculated that this large effect is not caused by the WT residue type, Q residue, but because of the mutant residue P and the location of mutation site. Because of that we will alter the corresponding |ΔΔ*G*_*k*_| with respect to each of the 4^th^ flags by applying the following formula:

$$\left|\Delta \Delta G_{k}^{altered}\right|(forP_{i}set)=\left\{\begin{array}{c} \frac{2}{3}\cdot \sum_{j=1,i<>j}^{4}P_{j}\cdot \left|\Delta \Delta G_{k}\right|,\left|\Delta \Delta G_{k}\right|<1\\ \frac{2}{3}\cdot \sum_{j=1,i<>j}^{4}(1-P_{j})\cdot \left|\Delta \Delta G_{k}\right|,\left|\Delta \Delta G_{k}\right|\geq 1 \end{array}\right\}$$ (3)

where *P*_*j*_ stands for: *P*_1_ = *P*(*Q* → *any*), *P*_2_ = *P*(*any* → *P*), *P*_3_ = *P*(*COR*, *WT*), and *P*_4_ = *P*(*SUP*, *MT*). These alterations are done for each entry in sDB and for each set of flags. In the entry “k”, original |ΔΔ*G*_*k*_| is larger than 1kcal/mol and therefore in the particular case considered above the second row formula is applied. If the original experimental binding free energy change is smaller than 1kcal/mol, the first row formula is applied. To further quantify the applied alterations, we would like to point out that in the extreme case when all three probabilities are 0.5 (i.e. the initial statistical analysis of sDB shows that the type of mutation has equal chance to cause large and small effect), applying Eq (17) will result in no alteration (no change).

The resulting set of |ΔΔ*G* ^*altered*^| is termed altered dataset and subsequently was used to recalculate the probabilities P (Table 2 for residue types and Table 3 for the mutation location). The results are shown in Figs 3 and 4 as well. These probabilities and classifications will be used to improve the performance of SAAMBE method. Given a particular mutation (for example, *Q* → *P*) and its location at the interface (for example COR in WT and SUP in MT) we calculate the probability of the mutation to cause large effect as:

$$P=\frac{P\left(P\to any\right)+P\left(any\to A\right)+P\left(COR,\;WT\right)+P\left(SUP,\;MT\right)}{4}$$ (4)

**Table 2:** The probability of residues type to cause small/large effect while being in WT/MT positions based on weighted absolute value of the experimental change in binding free energy.

	WT_Ncases	P(X→any)_small	P(X→any)_large	MT_Ncases	P(any→X)_small	P(any→X)_large
**A**	88	0.58	0.42	923	0.64	0.36
**C**	7	0.86	0.14	45	0.58	0.42
**D**	112	0.49	0.51	68	0.49	0.51
**E**	194	0.53	0.47	68	0.47	0.53
**F**	44	0.61	0.39	88	0.64	0.36
**G**	96	0.27	0.73	59	0.31	0.69
**H**	40	0.53	0.48	46	0.57	0.43
**I**	52	0.58	0.42	52	0.44	0.56
**K**	201	0.65	0.35	80	0.43	0.58
**L**	133	0.34	0.66	63	0.43	0.57
**M**	25	0.76	0.24	50	0.52	0.48
**N**	158	0.68	0.32	56	0.50	0.50
**P**	99	0.70	0.30	42	0.19	0.81
**Q**	57	0.81	0.19	70	0.54	0.46
**R**	177	0.63	0.37	68	0.40	0.60
**S**	91	0.80	0.20	62	0.44	0.56
**T**	162	0.44	0.56	42	0.43	0.57
**V**	52	0.58	0.42	58	0.47	0.53
**W**	54	0.43	0.57	54	0.46	0.54
**Y**	199	0.30	0.70	47	0.51	0.49

**Table 3:** The probability of residues location to cause small/large effect while being in WT/MT positions based on weighted absolute value of the experimental change in binding free energy.

	WT_Ncases	P(Y,WT)_small	P(Y,WT)_large	MT_Ncases	P(Y,MT)_small	P(Y,MT)_large
**COR**	1013	0.34	0.66	875	0.34	0.66
**INT**	121	0.77	0.23	200	0.71	0.29
**RIM**	509	0.70	0.30	520	0.62	0.38
**SUP**	150	0.49	0.51	156	0.44	0.56
**SUR**	248	0.92	0.08	290	0.90	0.10

**Fig 5:**
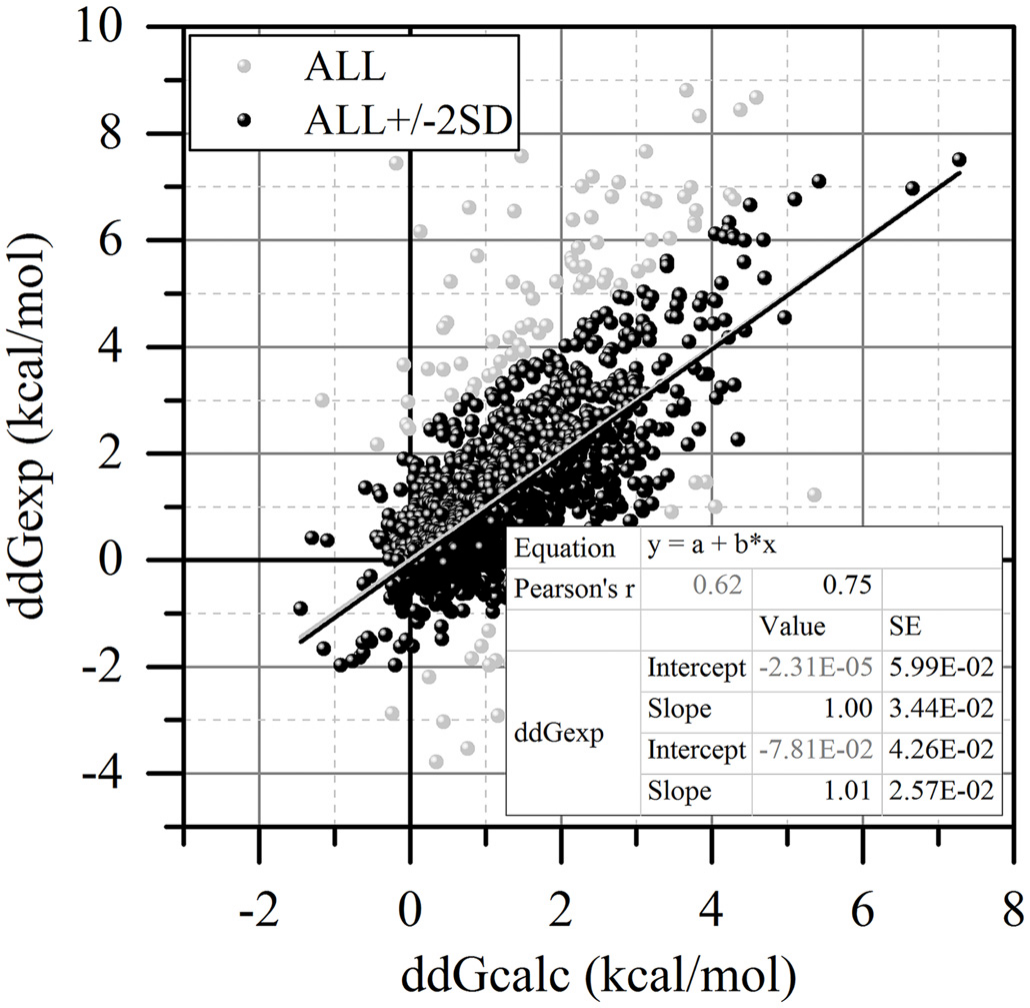
Correlation between experimental and calculated with SAAMBE approach data of change in binding free energy due to single point mutations for tDB (grey dots) and the one within ±2SD (black dots).

Thus, if P ≥ 0.5 the mutation is classified as a mutation expected to cause large change of the binding free energy. Otherwise, the mutation is expected to cause a small change. Thus, the final refinement of SAAMBE method is to take advantage of estimated probabilities. For each entry in the tDB we calculated the average probability P and split the database into tDB_small (P < 0.5) and tDB_large (P ≥ 0.5). For each of subsets we calculated the change in binding free energy (Eq (12)) and obtained the optimal coefficients of each energy terms in SAAMBE by multiple linear regression analysis. This resulted in two sets of SAAMBE coefficients (Table 1). For comparison we also provide the optimized weights and the correlation coefficient for the total tDB as well (Table 1). Comparing the weight coefficients in Table 1, one can see that there are some energy terms that are important for both subsets (such as EE, VE, SP, IE, entropy and Interface). Most of the mutations in the sDB_small are non-interfacial (for more than 30% of this subset the WT residue is located in the INT or SUR) and solvent exposed (~50% in RIM). Based on the magnitude of the weight coefficients, one can speculate that the changes of the binding free energy might be caused by the slight reorganization of the whole protein-protein complex that is reflected in the $\frac{\Delta \Delta SASA}{Interface}$ component energy term as well as the change in nonpolar component of salvation energy (*SN*). On the other hand most of the mutations in the sDB_large are located at the interface (95% are in COR, 5% in SUP area). In addition to other energy terms, for the cases of sDB_large, the change in hydrogen bonds network and the change in hydrophobicity also play significant roles. Thus, adding such features into the SAAMBE protocol, namely having different weight coefficients in the SAAMBE formula for mutations expected to cause small/large effect on the binding free energy change, increases the correlation coefficient from 0.575 to 0.624 (see Table 1 and Fig 5).

### Algorithm performance

To evaluate the performance of the SAAMBE method we analyzed six ROC parameters. The results obtained by the SAAMBE algorithm were compared with those calculated by FoldX and BeAtMuSiC methods for the same tDB. According to the Table 4 the number of true positive predictions is twice as high for the SAAMBE as for the other two algorithms. The total number of false predictions is much smaller for SAAMBE. This indicates that SAAMBE outperforms FoldX and BeAtMuSiC by all six ROC parameters using tDB as a benchmark. In terms of numbers, SAAMBE benchmarking results in: sensitivity, or true positive rate, (0.87); NVP, or negative predictive value, (0.84); method accuracy (0.9); and MCC (0.84). This proves that SAAMBE can predict with high accuracy not only the direction of the change in binding free energy, but also its magnitude.

**Table 4:** ROC parameters.

	SAAMBE	FoldX	BeAtMuSiC
**tn**	239	292	235
**fn**	47	133	141
**tp**	320	192	175
**fp**	5	11	7
**sensitivity**	0.872	0.591	0.554
**specificity**	0.980	0.964	0.971
**precision**	0.985	0.946	0.962
**NVP**	0.836	0.687	0.625
**accuracy**	0.915	0.771	0.735
**MCC**	0.836	0.592	0.555

### Mutations involving special cases

SAAMBE method was developed and optimized to predict the change of binding free energy for a broad range of mutation types. In this subsection we would like to address the question of how SAAMBE protocol can handle special cases: a) when the bulky residue is substituted with the small one; b) when the MT residue is Ala, which is typically used for protein “hot-spot” prediction; and c) the ability to accurately predict the effect of mutations being in a particular location. We will also compare our results with those delivered from FoldX and BeAtMuSic methodologies (see Table 5).

**Table 5:** Performance of SAAMBE, FoldX and BeAtMuSiC in predicting of "large-to-small" and ALA-scanning mutation as well as the mutation in specific location.

		SAAMBE	FoldX	BeAtMuSiC
**Large-to-Small (173)**	R	**0.489**	0.402	0.412
	RMSD	1.429	1.500	1.492
	y-Intercept	0.328	0.878	0.343
	Slope	0.692	0.528	0.632
**ALA-scanning (577)**	R	**0.488**	0.376	0.356
	RMSD	1.295	1.374	1.386
	y-Intercept	0.268	0.722	0.405
	Slope	0.695	0.532	0.587
**COR,SUP (807)**	R	**0.461**	0.273	0.305
	RMSD	1.733	1.879	1.860
	y-Intercept	0.351	1.580	1.197
	Slope	0.813	0.223	0.544
**RIM,SUR,INT (518)**	R	**0.478**	0.159	0.282
	RMSD	1.009	1.134	1.103
	y-Intercept	-0.024	0.493	0.194
	Slope	1.023	0.329	0.735

#### “Large-to-small” residue substitution

For this analysis we consider large WT residues to be R, F, W and Y and the set of small residues in MT comprised of A, G and S [24]. This results in 173 cases in the tDB. SAAMBE shows the highest correlation coefficient (0.49) (Table 5). It is interesting to note that although BeAtMuSiC method results in the same correlation coefficient as FoldX, the linear fits (slope and y-intercept) are very similar to those of SAAMBE.

#### Ala substitutions

Alanine is a small hydrophobic residue that is typically used to identify “hot-spots” of proteins and protein-protein interactions. Thus one may speculate that if a residue is mutated to Ala and causes large change in binding free energy, the WT residue plays important role in the binding process. For the 577 cases in the tDB involving the mutations to Ala, SAAMBE again results in the best correlation coefficient (0.49) comparing to FoldX (0.38) and BeAtMuSiC (0.36). Location of the mutation site.

For this type of analysis we considered two sets of locations where mutation can occur. The first location set is made of COR and SUP areas and represents the most buried part of the interface. As seen from Fig 4 mutations located in these two regions are expected to cause large change in the binding free energy. The second location set is made of SUR, INT and RIM regions. These regions are much more accessible from the water phase as compared with the COR and SUP and it is expected that mutations occurring in the second region will cause small changes of the binding free energy (Fig 4). The results of the benchmarking are shown in Table 5. It can be seen that SAAMBE algorithm outperforms FoldX or BeAtMuSiC.

### Time of calculations

**Fig 6:**
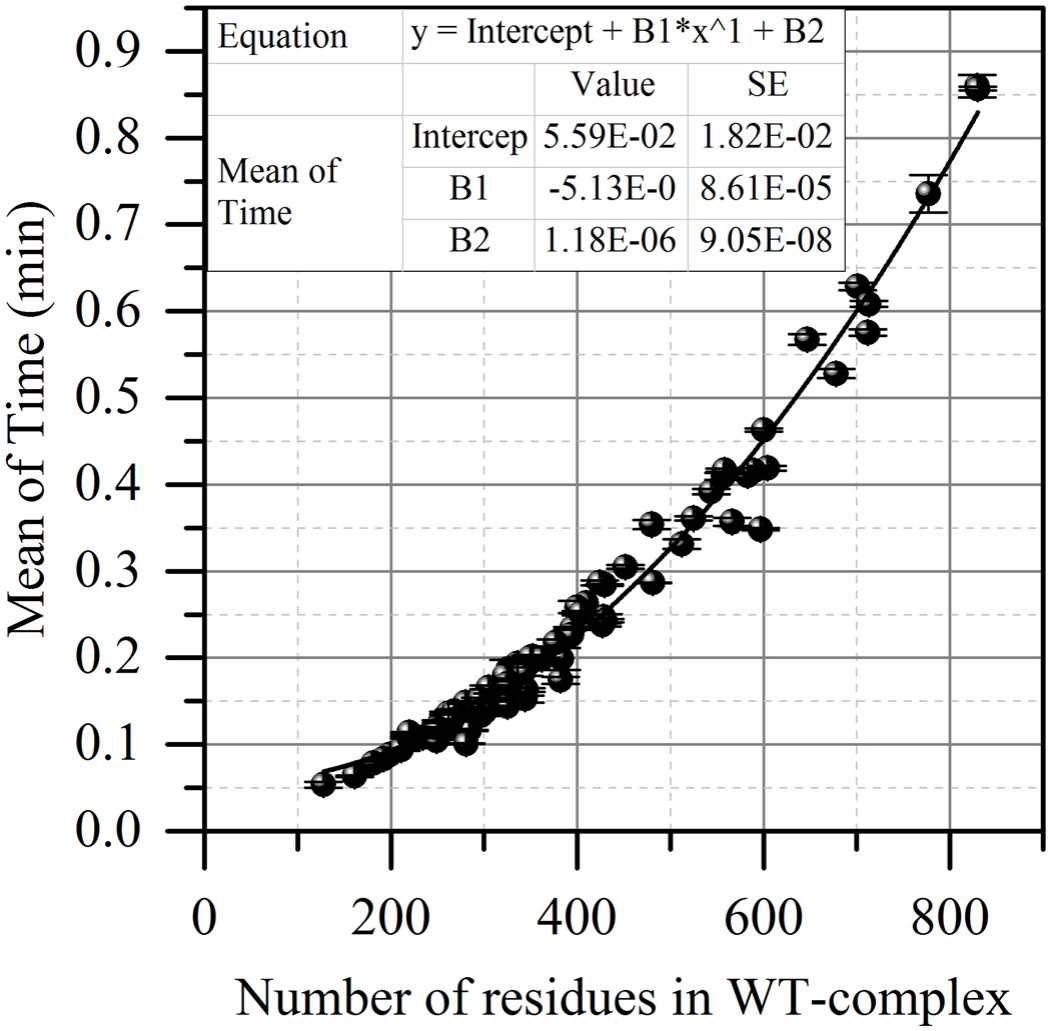
The dependence of the mean time of the algorithm execution from the total number of residues in the WT-complex.

One of the main considerations in developing SAAMBE algorithm was the requirement of the predictions to be made in reasonable time. We tested the time of the algorithm execution for all entries in sDB. The average time was 0.21953 min for one mutation calculation (SE = 0.00316min) when employing 16 nodes for WT- and MT-complexes minimization and single node for the rest of calculation on Clemson University Palmetto Supercomputer (http://citi.clemson.edu/palmetto/). We also analyzed the effect of particular parameters such as the number of residues in the complex and the largest dimension for the WT-complex on the time of calculations. It was found that the shape of the protein has no impact on the time of calculations. However the total number of residues in the complex affects the total calculation time (Fig 6). One can see that the dependence of time of algorithm execution vs the total number of residues in the WT-complex can be described with polynomial (second power) function (R = 0.99, 81 points). The free coefficient is 5.59E-2 min, the linear and quadratic weights are -5.13E-5 min and 1.18E-6 min respectively.

### Conclusions

In this work we described a development of a method, the SAAMBE method, to predict the binding free energy changes caused by single mutations. In developing the method, we were particularly interested in using structural information in conjunction with other types of information. This was motivated by the goal to deliver not only correct predictions of the energy changes, but also to be able to offer an explanation of the reason for the effect. Thus, the algorithm has structure-related components, such as hydrogen bonds, interface area, and interface area change. In addition, the MM/PBSA-based components indicate the importance of the direct interactions to the predicted energy changes. Thus, for any predictions, one can qualitatively describe what the major driving effects are. Furthermore, these energy changes can be compared with experimentally observed quantities or with observation delivered from more rigorous methods as FEP or IT.

The essential component of this investigation and development was the treatment of the plausible conformational and ionization changes induced by the binding. It is well understood that the binding introduces conformational and ionization changes, in some cases very small (almost rigid body binding like lock and key), in other cases large conformational changes (induced fit mechanism) [37–40]. Some of these changes occur far away from the binding interface and typically involve surface groups [37–40]. However, modeling such conformational changes is not trivial, especially if one aims at relatively fast predictions. Our attempts to model the plausible conformational changes induced by the binding via relatively short MD simulations were unsuccessful. Perhaps longer MD simulations complemented with enhanced sampling techniques are needed, but this is computationally too costly for large-scale predictions.

Instead of explicit modeling of conformational and ionization changes induced by the binding, we extend our previous approach to model them in electrostatic calculations via amino acid specific dielectric constant [36]. The motivation is based on the understanding that charged residues have the largest effect on electrostatic potential via their charges and ability to adopt different rotamers in response to the electrostatic field or to change their ionization states. Therefore, charged residues should be modeled with a large dielectric constant. Similarly, polar residues are the second in the list, since they have strong dipole moment and can participate in various hydrogen bonds. The rest of the amino acids, mostly hydrophobic residues, do not have many polar atoms and are typically buried in protein interior (and therefore packed and not able to sample different rotamers) and should be modeled with low dielectric constant [36] (for more details see Figs A and B in S1 Text). Indeed, the development reported in this work confirmed the applicability of such an approach and significantly improved the performance of SAAMBE method. Using DelPhi capability to assign different dielectric constants for different amino acids, we demonstrated that charged, polar and other residues should be modeled with dielectric constants 9, 8, and 7, respectively. This proves to be very effective and computationally inexpensive approach to mimic conformational flexibility in the framework of continuum electrostatics.

The SAAMBE method is a formula made of linear combination of terms: energy, empirical or statistical terms. The quantities or the physical phenomena described by some of them partially overlap, which can be considered as double-counting. However, the statistical analysis (p-values in Table 1) indicates that their values are acceptable (for more details see Tables A-D in S1 Text). Thus, while there is partial overlap for some terms, because of the simplifications made in modeling these phenomena, different terms capture different components of the process and thus they are almost independent.

The weight coefficients in the SAAMBE method were optimized against experimentally determined binding free energy changes of the tDB set. Therefore, the prediction accuracy depends on the training dataset and cases to be tested. It is anticipated that if the newly identified cases to be predicted by SAAMBE protocol do not deviate much from the cases in sDB/tDB, the predictions will be quite accurate. However, it is quite possible as well, that a new case is very different from the cases in sDB/tDB and then the prediction may not be accurate. We plan to continue enriching sDB/tDB and re-adjust the weight coefficients (if needed) of SAAMBE method and taking advantage of the computational cost to implement SAAMBE into a webserver.

## Methods

### Construction of data sets

We compiled a dataset, containing experimentally measured values of changes in binding free energy of protein-protein complexes due to single amino acid substitutions, by combining three sets of data mentioned in the following references: [25], [23] (Ala scanning database), and Skempi database [17]. To avoid the redundancy, all entries in the initially combined data set were screened to identify identical cases and only one representative was retained in the dataset. Then the dataset was further purged with respect to the experimental value of the binding free energy change. Thus, when several experimental values were available for the same mutation in the same protein-protein complex, and the experimental data variation was smaller than 1.5 kcal/mol (the threshold was empirically selected), the entries were fused and the averaged value for the change of the binding free energy was used. If the variation was larger than 1.5kcal/mol, the entry was deleted. Furthermore, mutations located in structurally disordered protein segments (missing coordinates in the PDB file) were removed from the dataset as well.

As a result, the final compiled dataset was comprised of 81 different proteins with the total of 2041 single point mutations. This dataset will be used for the statistical analysis of experimental data and will be referred to as sDB hereafter. However, to construct a dataset for training and testing, we further pruned the entries to remove all structures having heteroatoms (crystallographic water molecules were not considered heteroatoms). The motivation was that while some compounds listed in the heteroatoms section of PDB file may be biologically important, the vast majority of them are crystallographic artifacts (as ions for example). Thus, the resulting pruned database (tDB) consists of 1326 single point mutations from 43 proteins. Both datasets are available for download from (compbio.clemson.edu/databases/sDB,tDB.xlsx).

### Location of mutated residues

We assigned the location of mutated residues in the protein-protein complex based on five categories (COR, SUP, RIM, INT and SUR) as previously described [41] by computing the relative solvent accessible surface area (SASA) (the ratio between SASA of a residue in protein and in water (*rSASA*); *rSASA* = 1 corresponding to totally exposed residue in the protein) of the residue in the monomeric (*rSASAm*) and complex (*rSASA*c) states, as well as their mutual difference (Δ*rSASA* = *rSASAm* − *rSASAc*). Thus residues are considered to be at the interface if they are in COR, SUP and RIM regions; and are away from the interface if they are in SUR and INT regions. RIM and SUR locations indicate that the residue is exposed to the water solvent when the complex is formed. The parameters of each location types are provided in Table 6. The solvent accessible surface area of a residue was calculated with NACCESS software [42].

**Table 6:** Parameters of the residues location types in the protein-protein complex.

Location	Interface	Solvent exposure	rSASAm	rSASAc	ΔrSASA
COR	Yes	No	> 25%	< 25%	> 0
SUP	Yes	No	< 25%	< 25%	> 0
RIM	Yes	Yes	any	> 25%	> 0
INT	No	No	any	< 25%	= 0
SUR	No	Yes	any	> 25%	= 0

### Simulation protocol

The initial crystal structures of the protein-protein complexes were obtained from the Protein Data Bank (PDB) [43]. Biological units were retrieved and only chains that belonged to the binding partners were retained for further calculations. Since the initial crystal structures might have regions with missing coordinates, we used the *profix* module from Jackal package to rebuild these regions [44]. It was done using default parameters and selecting “heavy atoms model” option. At the next step we applied the *scap* module from the same Jackal package to substitute wild-type residue with the mutant to generate the mutant (MT)-complex. To eliminate inconsistency that might be associated with applying *scap* software we also substituted wild-type residue with the same residue using *scap* to generate the wild-type (WT)-complex. To run *scap* we applied the following parameters: (a) CHARMM22 force field parameters, (b) large side-chain Jackal rotamer library was selected for the side-chain refinement, and (c) predictions were made applying the *scap* option utilizing 3 initial structures. Once the WT and MT structures were generated, the missing hydrogen atoms were added to the structures with VMD software (version 1.9.1, topology file from CHARMM27 force field) [45]. Both WT- and MT-complexes were subjected for independent structural refinement by NAMD (version 2.9, CHARMM27 force field parameters) [46]. For the minimization procedure we used Generalized Born implicit solvent model (GBIS), implemented in NAMD. The dielectric constant of the implicit solvent was set to be 80, and 1 for the protein (various protein dielectric constants were tested—see Result section). We used quick N-steps (optimum value for N was found to be 5000, see Result section) conjugate gradient algorithm implemented in NAMD to obtain the relaxed configuration with optimized geometric and steric clashes. The energy-minimized structures of WT and MT complexes were used to calculate all energy components for both the complex (bound molecules) and monomers (unbound molecules). Typically such an approach is refereed as to rigid body approach.

### Binding energy calculations

The binding free energy was calculated based on modified MM/PBSA method combined with knowledge-based energy terms. The individual energy terms are combined via weighted linear function, typically referred as to linear interaction energy (LIE) formula or scoring function. Here we chose to term the method as Single Amino Acid Mutation based change in Binding free Energy (SAAMBE) method. It has two major components: (a) energy components calculated with MM/PBSA technique and (b) knowledge-based terms delivered from statistical analysis of entries in sDB. In developing the SAAMBE protocol, we first define the terms (*E*) that will be used in SAAMBE protocol as follows:

$$\Delta \Delta E=(E_{{}_{AB}}^{MT}-E_{{}_{A}}^{MT}-E_{{}_{B}}^{MT})-(E_{{}_{AB}}^{WT}-E_{{}_{A}}^{WT}-E_{{}_{B}}^{WT})$$ (5)

where “AB” stands for the protein complex and “A” and “B” notations correspond to the unbound monomers. The superscripts WT and MT refer to wild type and mutant, respectively. Thus, Eq (5) provides the difference of the contribution *(ΔΔE*) of a particular energy term *E* to the change of the binding free energy caused by a mutation. It should be reiterated that unbound monomer structures were taken from the complex, thus no structural changes are considered to be caused by the binding. In addition, it should be clarified that these terms (*E*) could be potential energies as in case of MM/PBSA delivered terms, or could be an estimation of the entropy change associated with the binding, or could be a term delivered from statistical analysis, for example. Thus their absolute values and dimensionalities vary drastically, but these differences are absorbed by the weight coefficients in the SAAMBE formula. Since weight coefficients in SAAMBE formula are optimized to result in best match against experimentally determined binding free energy changes, the quantity delivered by SAAMBE formula is termed binding free energy change as well (ΔΔ*G*). Below we describe separately the MM/PBSA and the knowledge-based developments of SAAMBE method.

### The MM/PBSA-based component of the SAAMBE method

The MM/PBSA-based component of the SAAME method is a linear combination of five weighted energy terms:

$$\Delta \Delta G^{MM/PBSA}=w_{0}+w_{1}\cdot \Delta IE+w_{2}\cdot \Delta \Delta EE+w_{3}\cdot \Delta \Delta VE+w_{4}\cdot \Delta \Delta SP+w_{5}\cdot \Delta \Delta SN$$ (6)

Where Δ*IE* is the change of the total internal energy of complexes. Other energy terms are: ΔΔ*EE* is the change of Coulomb energy, ΔΔ*VE* is the change of van der Waals (vdW) energy, ΔΔ*SP* and ΔΔ*SN* are the changes of polar and nonpolar components of solvation energy calculated with Eq (5). *w*_*i*_ are the weight coefficients which will be optimized against experimental data in tDB. Below we describe the details of calculations of each energy term in Eq (6).

*ΔIE* component was calculated as the energy difference of all internal energy terms (bonded potential, angle potential, and torsion potentials) of the WT and MT complexes. Strictly speaking, the change of the internal energy should be calculated with Eq (5), but since the bound and unbound structures in SAAME protocol are the same, using Eq (5) will result in zero change of the internal energy. Because of that *ΔIE* is taken as the difference of the internal energy of complexes only. Obviously this is inconsistent with MM/PBSA methodology and is uninformative thermodynamic quantity, but was accepted since the benchmarking against experimental data showed that adding such energy term in Eq (6) improves the quality of the predictions (see Results section). The internal energy was calculated with NAMD.

*ΔΔVE* were calculated with the NAMD program using the WT and MT complexes and separated monomers to deliver the terms described in Eq (5). It was done by taking the structures on the monomers from already energy-minimized structure of the corresponding complex. Then, each complex, WT and MT, and each separate monomer, WT and MT, were subjected to one step minimization with NAMD to obtain the corresponding vdW energies.

*ΔΔEE* and *ΔΔSP* energies were calculated with DelPhi software [47] with the following parameters: linear Poisson-Boltzmann solver, scale 1 grids/Å, perfil 70% and external dielectric constant 80. The choice of the value of internal dielectric constant requires explanation. As it was mentioned above, SAAMBE protocol is rigid body protocol, i.e. the structures of bound and unbound monomers are identical. However, binding is expected to induce small or large structural changes, which are not taken into account in the model explicitly. In the past, we demonstrated that the effects of structural changes on the electrostatic energy can be mimicked by appropriate dielectric constant by assigning specific dielectric constant values to different protein regions [48]. Although our previous analysis was done for folding free energy changes caused by mutations [48], the same principle should be valid for protein binding free energy modeling. Thus, in the development of SAAMBE protocol, the protein interior was considered to be inhomogeneous and inhomogeneity was modeled via three different dielectric constants (ε_1_, ε_2_ and ε_3_). Thus all charge groups (Asp, Glu, Lys, Arg and His) were modeled withε_1_, all polar groups (Ser, Thr, Asn, Gln and Tyr) with ε_2_ and the rest of amino acids with ε_3_. The values of these residue-specific dielectric constants were systematically varied as discussed in the Results section. DelPhi allows for such multi-dielectric modeling [49]. The polar component of solvation energy was calculated via “corrected reaction field energy” module of DelPhi, for both the complexes and separated monomers and then applying Eq (5) to obtain the difference (*ΔΔSP*). The Coulombic energies were also calculated with DelPhi for the complexes and separated monomers and the applying Eq (5) to deliver *ΔΔEE*. It should be mentioned that the calculated *ΔΔEE* is not the standard *ΔΔEE* in MM/PBSA approaches. It is well-known that electrostatic interactions between covalently bound atoms are already taken into consideration via internal energy terms and should not be part of *ΔΔEE* (this is taken care in all MD packages). However, taking *ΔΔEE* from the NAMD output resulted in worse performance of SAAMBE method (as judged by fitting the predictions against experimental data) and this was the reason to accept such an inconsistency.

The nonpolar component of the solvation energy was calculated via linear formula with respect to SASA of the protein and protein complexes (Eq (7)). The SASA was calculated with NACCESS software [42] and the corresponding coefficients in Eq (7) were re-distributed in Eq (6) as: *α* takes part in the weight *w*_5_ while *β* is absorbed in the free coefficient *w*_0_.

SN=α⋅SASA+β (7)

### The knowledge-based components of the SAAMBE method

The knowledge-based components were calculated according to the formula:

$$\Delta \Delta G^{KB}=w_{6}\cdot \Delta \Delta S+w_{7}\cdot \Delta \Delta HYDR+w_{8}\cdot \Delta HB+w_{9}\cdot Interface^{MT}+w_{10}\cdot \frac{\Delta \Delta SASA}{Interface^{MT}}$$ (8)

where five additional terms were taken into account: entropy (*S*), hydrophobicity (*HYDR*), hydrogen bonds (*HB*), interface area of the MT-complex (*Interface* ^*MT*^), and the change of the interface area caused by the mutation normalized to the total interface of the MT-complex ($\frac{\Delta \Delta SASA}{Interface^{MT}}$).

The entropy of the residues in complex and in the corresponding monomers was estimated based on an empirical formula developed in this work. It is based on the maximal number of side chain rotamers (R) taken from Ref. [50]. The maximum number of rotamers for each residue is provided in Table 7. However, we assume that the ability of given amino acid side chain to sample its maximum number of rotamers will depend on its exposure to the surface, i.e. fully exposed residue with relative SASA (rSASA) equal to one will be able to access all rotamers, while completely buried one (rSASA = 0) will be completely rigid adopting a particular rotamer. Having in mind that entropy is proportional to the logarithm of states (in our case rotamers), the corresponding formula for this particular residue is:

S=ln[rSASA⋅(R−1)+1] (9)

Eq (9) is applied to the complexes and individual monomers and the Eq (5) is used to deliver *ΔΔS*.

**Table 7:** The maximum number of rotamers and hydrophobicity of the residues.

	A	C	D	E	F	G	H	I	K	L	M	N	P	Q	R	S	T	V	W	Y
*R*	1	3	18	54	18	1	36	9	81	9	27	36	2	108	81	3	3	3	36	18
*H*	0.2	-0.2	1.2	1.01	-1.1	0	0.57	-0.3	1	-0.6	-0.2	0.4	0.5	0.6	0.8	0.1	0.1	0.1	-1.9	-0.9

The term accounting for the hydrophobicity was modeled using Wimley-White (*H*) hydrophobicity scale [51] (see Table 7) (different hydrophobicity scales were tested, but were found to perform worse in benchmarking of SAAMBE protocol against experimental data). The empirical formula was developed in this work assuming the following: an amino acid contributes to the hydrophobicity depending on its *rSASA*. For example, a residue being exposed to the water phase will have large contribution to *HYRD* while practically zero if buried inside the protein. Having in mind that *H*_*j*_ indexes have opposite signs for hydrophobic and hydrophilic amino acids, such a formulation qualitatively describes the physical basis of the hydrophobic effect. The corresponding formula is:

$$HYDR=\sum_{j=1}^{N}H_{j}\cdot rSASA_{j}$$ (10)

As above, the formula is applied to the corresponding complexes and separate monomers and then Eq (5) to deliver *ΔΔHYDR*.

The impact of the mutations on the formation of hydrogen bonds (*HB*) was taken into account as well. We computed the number of *HB* for WT ($\sum HB_{A-A}^{WT}\text{ and }\sum HB_{B-B}^{WT}$) and MT ($\sum HB_{A-A}^{MT}\text{ and }\sum HB_{B-B}^{MT}$) monomers and at the same time the number of hydrogen bonds that were formed between monomers in the corresponding complex ($\sum HB_{A-B}^{MT}\text{ and }\sum HB_{A-B}^{WT}$). The first class represents the intra-monomer bonds, and the second inter-monomer bonds. It is assumed that intra-monomer *HB* change resulting in more *HB* in the mutant, Δ*HB* > 0, will make MT monomers more stable than the WT, and thus might decrease binding free energy. In contrast, Δ*HB* >0 of inter-monomer *HB* is expected to increase binding affinity of the MT compared with WT. Because of such considerations, the effect of *HB* on the binding free energy change was calculated as:

$$\Delta HB=(\sum HB_{A-B}^{MT}-\sum HB_{A-A}^{MT}-\sum HB_{B-B}^{MT})-(\sum HB_{A-B}^{WT}-\sum HB_{A-A}^{WT}-\sum HB_{B-B}^{WT})$$ (11)

where the HB was counted as cases involving two atoms oxygen acceptor and hydrogen (except the nonpolar C_α_ and C_β_ hydrogen atoms, HA and HB) atoms located at distance shorter than 2.4 Å. Since the nitrogen acceptor is much weaker than oxygen, for simplicity it was not considered. Similarly the geometry of the hydrogen bond was not taken into consideration. Only polar (S, T, N, Q, Y) and charge (R, H, K, D, E) amino acids were taken into account.

Our previous work [24] indicated that the surface area of the interface in the MT-complex is an important factor in predicting binding free energy changes. Because of that, it is included in this protocol as well and was calculated as the difference in SASA of complex and the sum of each of its parts.

ΔΔ*SASA* was calculated with Eq (5) as the difference in SASA of the complex and monomeric states of MT and WT.

### Combining MM/PBSA-based and knowledge-based terms, the final SAAMBE formula is

$$\begin{array}{l} \Delta \Delta G=\Delta \Delta G^{MM/PBSA}+\Delta \Delta G^{KB}=w_{0}+w_{1}\cdot \Delta IE+w_{2}\cdot \Delta \Delta EE+w_{3}\cdot \Delta \Delta VE+w_{4}\cdot \Delta \Delta SP+w_{5}\cdot \Delta \Delta SN+\\ +w_{6}\cdot \Delta \Delta S+w_{7}\cdot \Delta \Delta HYDR+w_{8}\cdot \Delta HB+w_{9}\cdot Interface^{MT}+w_{10}\cdot \frac{\Delta \Delta SASA}{Interface^{MT}} \end{array}$$ (12)

### Receiver operating characteristics (ROC)

In order to quantify the performance of our algorithm and compare it with other methods we evaluated the calculated and experimental values of change in binding free energy due to single point mutation and assigned one of four flags for each entry in the tDB: true positive (tp), true negative (tn), false positive (fp), or false negative (fn). The explanation of the assignment procedure is provided in the Table 8.

**Table 8:** Four outcomes of calculation based on the ability of the algorithm to predict the ΔΔ*G*.

	true	false
**positive**	|ΔΔ*G_calc_*|≥1.5 & |ΔΔ*G_exp_*|≥1.5 & *sing*(ΔΔ*G_calc_*) = *sing*(ΔΔ*G_exp_*)	|ΔΔ*G_calc_*|≥1.5 & |ΔΔ*G_exp_*|<0.5
**negative**	|ΔΔ*G_calc_*|<0.5 & |ΔΔ*G_exp_*|<0.5	|ΔΔ*G_calc_*|<0.5 & |ΔΔ*G_exp_*|≥1.5

The quality of the predictions was described by six parameters: accuracy, precision, sensitivity, specificity, negative predictive value (NPV) and Matthews correlation coefficient (MCC) [52,53]:

$$\text{accuracy}=\frac{\text{tn + tp}}{\text{tn+tp+fn+fp}}$$ (13)

$$\text{sensitivity}=\frac{\text{tp}}{\text{tp+fn}}$$ (14)

$$\text{specificity}=\frac{\text{tn}}{\text{tn+fp}}$$ (15)

$$\text{presicion}=\frac{\text{tp}}{\text{tp+fp}}$$ (16)

$$\text{NPV}=\frac{\text{tn}}{\text{tn+fn}}$$ (17)

$$MCC=\frac{\text{tp}\cdot \text{tn + fp}\cdot \text{fn}}{\sqrt{\text{(tp+fp)}\cdot \text{(tp+fn)}\cdot \text{(tn+fp)}\cdot \text{(tn+fn)}}}$$ (18)

### Speed performance

One of the goals of SAAMBE development is to develop fast algorithm capable of large-scale calculations. Thus, the execution time is an important component of the investigation. The execution time was monitored as a function of the number of amino acids in the corresponding complex (sequence length) and as a function of the geometrical shape of the complex (monitored via the largest dimension of WT complex).

### Statistical analysis

To verify the agreement between experimental and predicted values of the change of binding free energy due to single point mutation we calculated the Pearson correlation coefficient. In the paper all reported correlation coefficients were significantly different from zero with p-value smaller than 0.01.

We also performed five-fold cross validation test for the tDB. It was done by randomly partitioning the tDB into five subgroups of approximately equal size. Each combination of four subgroups was used for training, while the fifth—for testing the model. Then correlation coefficients were averaged over different cross-validated sets.

## Supporting Information

S1 Text **A**—**Distribution of the RMSD within charged (CRG: Arg, Asp, Glu, Hse, Lys, blue); polar (PLR: Asn, Gln, Ser, Thr, Tyr, orange) and other (OTR, green) groups of residues.** RMSD was estimated based on the deviation of the last heavy atom in a side chain of the residue in the protein-protein complex and in unbound part. Both protein-protein complex and its each partner were minimized for 5000 steps in NAMD. The inserted graph illustrates the average RMSD of the residues within CRG, PLR and OTR groups for WT (dark grey) and MT (light grey) structures. The analysis was performed for all entries in tDB (see manuscript for details); **B**–The distribution of the change in RMSD of 1) CRG and PLR residues (orange) and 2) CRG and OTR residues (green) for WT (solid line, open circles) and MT (dash-dot line and solid circles) structures calculated for each case in tDB; Table A—The standardized weights of significant energy terms in predicting the change in binding free energy due to single amino acid substitution; Table B—Variance Inflation Factor calculated based on the Pearson’s correlation coefficient; Table C—Variance Inflation Factor calculated based on the Spearman’s correlation coefficient; Table D—Variance Inflation Factor calculated based on the Kendall’s correlation coefficient.
(DOCX)
