# Supplementary material for: Predicting Binding Free Energy Change Caused by Point Mutations with Knowledge-Modified MM/PBSA Method
Source: PLoS Comput Biol. 2015 Jul 6;11(7):e1004276. doi: 10.1371/journal.pcbi.1004276 (PMC4492929; doi:10.1371/journal.pcbi.1004276)
Supplement: S1 Text — A—Distribution of the RMSD within charged (CRG: Arg, Asp, Glu, Hse, Lys, blue); polar (PLR: Asn, Gln, Ser, Thr, Tyr, orange) and other (OTR, green) groups of residues. RMSD was estimated based on the deviation of the last heavy atom in a side chain of the residue in the protein-protein complex and in unbound part. Both protein-protein complex and its each partner were minimized for 5000 steps in NAMD. The inserted graph illustrates the average RMSD of the residues within CRG, PLR and OTR groups for WT (dark grey) and MT (light grey) structures. The analysis was performed for all entries in tDB (see manuscript for details); B–The distribution of the change in RMSD of 1) CRG and PLR residues (orange) and 2) CRG and OTR residues (green) for WT (solid line, open circles) and MT (dash-dot line and solid circles) structures calculated for each case in tDB; Table A—The standardized weights of significant energy terms in predicting the change in binding free energy due to single amino acid substitution; Table B—Variance Inflation Factor calculated based on the Pearson’s correlation coefficient; Table C—Variance Inflation Factor calculated based on the Spearman’s correlation coefficient; Table D—Variance Inflation Factor calculated based on the Kendall’s correlation coefficient. (DOCX) [file pcbi.1004276.s001.docx]

**Supplementary material**

1. **Conformational changes induced by the binding. Implication for dielectric constant value.**

In this work we use different dielectric constants for charged (CRG), polar (PLR) and other (OTR) residues and claim that CRG should be modeled with large dielectric constant value (ε=9), PLR with smaller one (ε=8) and the smallest dielectric constant value (ε=7) should be assigned to OTR. These dielectric constant optimal values were obtained via multiple regression analysis against experimental data of the binding free energy changes, not by analyzing the conformational changes caused by the binding. As it was pointed out in the manuscript, the SAAMBE method is a rigid-body method that keeps the structures of unbound monomers in the same conformation as in the complex, but uses optimized dielectric constant values to mimic the effects of conformational changes. To probe that CRG, PLR and OTR resides undergo different conformational changes upon the binding, we carried out the following investigation.

Each protein, the wild type and the mutant, was subjected to the energy minimization procedure described in the Method section. The complex and unbound monomers were energy minimized separately. This resulted in different structures for bound and unbound monomers. The backbone conformations were found to be practically the same (for bound and unbound structures), however, the side chain orientations were different. To assess the magnitude of conformational changes of the side chains caused by the binding, we calculated the distance between bound and unbound position of the last heavy atom of the corresponding side chain. We grouped the results according to the residue type as CRG, PLR and OTR. The analysis was done for both the wild type (WT) and each mutant (MT). Fig. A illustrates the distribution of RMSDs within CRG, PLR and OTR groups for all cases in tDB. One can see that on average (inserted figure) CRG residues in a protein tend to have the largest conformational changes upon binding, followed by PLR and the smallest conformational changes are found for OTR groups. Fig. A shows the distribution of RMSDs and indicates the same trend – indeed the distribution of RMSDs of charged group has much longer tail than the other two classes and it is shifted to larger conformational changes.

**Figure A** – Distribution of the RMSD within charged (CRG: Arg, Asp, Glu, Hse, Lys, blue); polar (PLR: Asn, Gln, Ser, Thr, Tyr, orange) and other (OTR, green) groups of residues. RMSD was estimated based on the deviation of the last heavy atom in a side chain of the residue in the protein-protein complex and in unbound part. Both protein-protein complex and its each partner were minimized for 5000 steps in NAMD [[1](#_ENREF_1)]. The inserted graph illustrates the average RMSD of the residues within CRG, PLR and OTR groups for WT (dark grey) and MT (light grey) structures. The analysis was performed for all entries in tDB (see manuscript for details).

Further analysis of conformational changes was performed by subtracting other two distributions (PLR and OTR) from the distribution of CRG. Results are shown in Fig. B. The positive value indicates that the RMSD of CRG residues is larger than RMSD of PLR/OTR residues.

**Figure B** – The distribution of the change in RMSD of 1) CRG and PLR residues (orange) and 2) CRG and OTR residues (green) for WT (solid line, open circles) and MT (dash-dot line and solid circles) structures calculated for each case in tDB. The positive number indicates that the RMSD or the CRG residues is larger than RMSD of PLR/OTR one.

Thus, the results indeed indicate that CRG side chains undergo larger conformational changes caused by the binding. According to the Fig. B in the majority of cases the RMSD of the CRG residues is 0.1 Å larger than of PLR residues, and 0.2 Å – of OTR residues. This provides additional confidence that dielectric properties of CRG, PLR and OTR groups should be modeled with different dielectric constants, the largest being the dielectric constant of CRG groups, followed by PLR and OTR.

1. **Sensitivity analysis of the plausible correlations between the terms in the prediction formula.**

As pointed out in the manuscript, some of the terms in the prediction formula reflect similar phenomena and may not be needed. To address such a possibility we applied “backwards elimination” procedure: Thus, we initially included all terms in multiple regression analysis (MLRA), then eliminated the least significant one, and rerun MLRA until only significant (p<0.1) terms remained. The investigation confirmed that all terms described in Table A are significant and should be kept in the equation.

Table A – The standardized weights of significant energy (see Table 6 in Manuscript) terms in predicting the change in binding free energy due to single amino acid substitution.

|  | **Weights** | | |
| --- | --- | --- | --- |
|  | **tDB_small** | **tDB_large** | **tDB** |
| Free | -3.31E-01 | 2.08E-01 | -6.59E-03 |
| **$\Delta\Delta EE$** | 1.11E+00 | 1.73E+00 | 1.76E+00 |
| **$\Delta\Delta VE$** | 2.42E-01 | 3.22E-01 | 3.40E-01 |
| **$\Delta\Delta SP$** | 9.60E-01 | 1.64E+00 | 1.60E+00 |
| **$\Delta\Delta SN$** | 9.96E-02 |  |  |
| **$\Delta\Delta S$** | 9.68E-02 | 9.48E-02 | 9.81E-02 |
| **$\Delta\Delta HYDR$** |  | -1.40E-01 | -1.58E-01 |
| **$Interface$** | 8.22E-02 | 2.15E-01 | 1.87E-01 |
| **$\Delta IE$** | 1.11E-01 | 1.79E-01 | 2.26E-01 |
| **$\Delta HB$** |  | 5.96E-02 |  |
| **$\frac{\Delta\Delta ASA}{Interface}$** | 4.25E-04 |  | 2.36E-03 |
| **Ncases** | 612 | 714 | 1326 |

In addition, we carried out the following analysis: Variance Inflation Factor (VIF) is considered to be an effective parameter reflecting the degree of collinearity between two terms (components) and is determined by the correlation coefficient ($R$):

$$VIF=\frac{1}{tolerance}=\frac{1}{1-R^{2}}$$

Two terms are considered to be highly collinear when VIF is more than 10.

Assuming that all independent terms (energy terms) obey normal distribution, we calculated the Pearson’s correlation coefficient for each pairs as well as corresponding VIF. Table B shows that most of variables are linearly independent and can be considered for multiple linear regression formula. Only EE (Coulombic energy) and SP (polar component of solvation energy) have high collinearity:$VIF_{Pearson}(EE,SP)>35$, but this is well known fact because the SP originates from EE and dielectric boundary.

The normality test (Shapiro-Wilk, Kolmogorov-Smirnov, Lilliefors, Anderson-Darling, D’Agostini-K squared and Chen-Shapiro) for independent terms indicated that none of them obeys normal distribution, probably due to the limited number of cases/proteins in database. Keeping this in mind we performed non-parametric correlation analysis between each pairs of independent terms. The data for Speanman’s and Kendall’s correlation coefficients and correspondent VIF show that all terms are linearly independent and thus can be considered for MLRA Tables C and D).

**Table B** – Variance Inflation Factor calculated based on the Pearson’s correlation coefficient.

|  | **$\Delta\Delta EE$** | **$\Delta\Delta VE$** | **$\Delta\Delta SP$** | **$\Delta\Delta SN$** | **$\Delta\Delta S$** | **$\Delta\Delta HYDR$** | **$\Delta\Delta Interface$** | **$\Delta IE$** | **$\Delta\Delta HB$** |
| --- | --- | --- | --- | --- | --- | --- | --- | --- | --- |
| **$\Delta\Delta VE$** | 1.001 |  |  |  |  |  |  |  |  |
| **$\Delta\Delta SP$** | 35.34 | 1 |  |  |  |  |  |  |  |
| **$\Delta\Delta SN$** | 1.01 | 1.747 | 1.01578 |  |  |  |  |  |  |
| **$\Delta\Delta S$** | 1.031 | 1.341 | 1.0379 | 1.22 |  |  |  |  |  |
| **$\Delta\Delta HYDR$** | 1.044 | 1.101 | 1.05626 | 1.004 | 1.007 |  |  |  |  |
| **$Interface$** | 1.004 | 1.006 | 1.00476 | 1.004 | 1.044 | 1.00016 |  |  |  |
| **$\Delta IE$** | 1.007 | 1.046 | 1.0072 | 1.027 | 1.085 | 1.00276 | 1.0423 |  |  |
| **$\Delta HB$** | 1.011 | 1.001 | 1.01117 | 1.003 | 1 | 1.00701 | 1.0122 | 1.0005 |  |
| **$\frac{\Delta\Delta ASA}{Interface}$** | 1.007 | 1.001 | 1.00694 | 1.001 | 1.004 | 1.0028 | 1.1117 | 1.0037 | 1.0059 |

**Table C** – Variance Inflation Factor calculated based on the Spearman’s correlation coefficient.

|  | **$\Delta\Delta EE$** | **$\Delta\Delta VE$** | **$\Delta\Delta SP$** | **$\Delta\Delta SN$** | **$\Delta\Delta S$** | **$\Delta\Delta HYDR$** | **$\Delta\Delta Interface$** | **$\Delta IE$** | **$\Delta\Delta HB$** |
| --- | --- | --- | --- | --- | --- | --- | --- | --- | --- |
| **$\Delta\Delta VE$** | 1 |  |  |  |  |  |  |  |  |
| **$\Delta\Delta SP$** | 5.265 | 1.011 |  |  |  |  |  |  |  |
| **$\Delta\Delta SN$** | 1.022 | 1.692 | 1.0716 |  |  |  |  |  |  |
| **$\Delta\Delta S$** | 1.027 | 1.325 | 1.06834 | 1.308 |  |  |  |  |  |
| **$\Delta\Delta HYDR$** | 1.047 | 1.048 | 1.04076 | 1.005 | 1.009 |  |  |  |  |
| **$Interface$** | 1 | 1.002 | 1.0009 | 1 | 1.042 | 1 |  |  |  |
| **$\Delta IE$** | 1.004 | 1.064 | 1.00749 | 1.044 | 1.094 | 1.005 | 1.041 |  |  |
| **$\Delta HB$** | 1.024 | 1.001 | 1.02352 | 1.004 | 1 | 1.003 | 1.018 | 1.002 |  |
| **$\frac{\Delta\Delta ASA}{Interface}$** | 1.013 | 1.011 | 1.01051 | 1.026 | 1 | 1.002 | 1.182 | 1.005 | 1.005 |

**Table D** – Variance Inflation Factor calculated based on the Kendall’s correlation coefficient.

|  | **$\Delta\Delta EE$** | **$\Delta\Delta VE$** | **$\Delta\Delta SP$** | **$\Delta\Delta SN$** | **$\Delta\Delta S$** | **$\Delta\Delta HYDR$** | **$\Delta\Delta Interface$** | **$\Delta IE$** | **$\Delta\Delta HB$** |
| --- | --- | --- | --- | --- | --- | --- | --- | --- | --- |
| **$\Delta\Delta VE$** | 1 |  |  |  |  |  |  |  |  |
| **$\Delta\Delta SP$** | 2.449 | 1.006 |  |  |  |  |  |  |  |
| **$\Delta\Delta SN$** | 1.01 | 1.296 | 1.03284 |  |  |  |  |  |  |
| **$\Delta\Delta S$** | 1.012 | 1.157 | 1.03036 | 1.148 |  |  |  |  |  |
| **$\Delta\Delta HYDR$** | 1.021 | 1.02 | 1.01882 | 1.006 | 1.006 |  |  |  |  |
| **$Interface$** | 1 | 1.001 | 1.00029 | 1 | 1.017 | 1 |  |  |  |
| **$\Delta IE$** | 1.002 | 1.03 | 1.00356 | 1.021 | 1.043 | 1.002 | 1.018 |  |  |
| **$\Delta HB$** | 1.012 | 1.001 | 1.01192 | 1.002 | 1 | 1.002 | 1.009 | 1.001 |  |
| **$\frac{\Delta\Delta ASA}{Interface}$** | 1.053 | 1.004 | 1.03379 | 1.019 | 1.009 | 1.052 | 1 | 1.002 | 1.084 |

1. Phillips JC, Braun R, Wang W, Gumbart J, Tajkhorshid E, et al. (2005) Scalable molecular dynamics with NAMD. Journal of computational chemistry 26: 1781-1802.
